# Outer Membrane Vesicles From *Fusobacterium nucleatum* Switch M0-Like Macrophages Toward the M1 Phenotype to Destroy Periodontal Tissues in Mice

**DOI:** 10.3389/fmicb.2022.815638

**Published:** 2022-03-21

**Authors:** Gang Chen, Qiang Sun, QiaoLing Cai, HongWei Zhou

**Affiliations:** ^1^Shenzhen Stomatology Hospital (Pingshan), Southern Medical University, Shenzhen, China; ^2^Department of Oral and Maxillofacial Surgery, The First Affiliated Hospital of Zhengzhou University, Zhengzhou, China; ^3^Department of Stomatology, The First Affiliated Hospital of Xiamen University, Xiamen, China; ^4^Microbiome Medicine Center, Department of Laboratory Medicine, Zhujiang Hospital, Southern Medical University, Guangzhou, China; ^5^State Key Laboratory of Organ Failure Research, Southern Medical University, Guangzhou, China

**Keywords:** *Fusobacterium nucleatum*, outer membrane vesicles, periodontitis, macrophages, inflammation

## Abstract

Periodontitis is a chronic inflammatory oral disease that affects nearly 50% of all adults. *Fusobacterium nucleatum* (*F. nucleatum*) is known to be involved in the formation and development of periodontitis. Outer membrane vesicles (OMVs) harboring toxic bacterial components are continuously released during *F. nucleatum* growth and regulate the extent of the inflammatory response by controlling the functions of immune and non-immune cells in tissues. Macrophages are important immune cells in periodontal tissue that resist pathogen invasion and play an important role in the pathophysiological process of periodontitis. However, the role of the interaction between *F. nucleatum* OMVs and macrophages in the occurrence and development of periodontitis has not been studied. The purpose of this study was to clarify the effect of *F. nucleatum* OMVs on the polarization of macrophages and the roles of this specific polarization and *F. nucleatum* OMVs in the pathophysiology of periodontitis. The periodontitis model was established by inducing ligation in C57BL/6 mice as previously described. Micro-CT, RT-qPCR, hematoxylin-eosin (H&E) and tartrate acid phosphatase (TRAP) staining assays were performed to analyze the periodontal tissue, alveolar bone loss, number of osteoclasts and expression of inflammatory factors in gingival tissue. The changes in the state and cytokine secretion of bone marrow-derived macrophages (BMDMs) stimulated by *F. nucleatum* OMVs were observed *in vivo* by confocal microscopy, flow cytometry, Western blot and ELISA. Mouse gingival fibroblasts (MGFs) were isolated and then cocultured with macrophages. The effects of *F. nucleatum* OMVs on the proliferation and apoptosis of MGFs were analyzed by flow cytometry and lactate dehydrogenase (LDH) assays. The periodontitis symptoms of mice in the *F. nucleatum* OMVs + ligation group were more serious than those of mice in the simple ligation group, with more osteoclasts and more inflammatory factors (IL-1β, IL-6, and TNF-α) being observed in their gingival tissues. M0 macrophages transformed into M1 macrophages after the stimulation of BMDMs with *F. nucleatum* OMVs, and the M1 macrophages then released more inflammatory cytokines. Analysis of the coculture model showed that the MGF apoptosis and LDH release in the inflammatory environment were increased by *F. nucleatum* OMV treatment. In conclusion, *F. nucleatum* OMVs were shown to aggravate periodontitis, alveolar bone loss and the number of osteoclasts in an animal model of periodontitis. *F. nucleatum* OMVs promoted the polarization of macrophages toward the proinflammatory M1 phenotype, and the inflammatory environment further aggravated the toxicity of *F. nucleatum* OMVs on MGFs. These results suggest that M1 macrophages and *F. nucleatum* OMVs play roles in the occurrence and development of periodontitis.

## Graphical Abstract

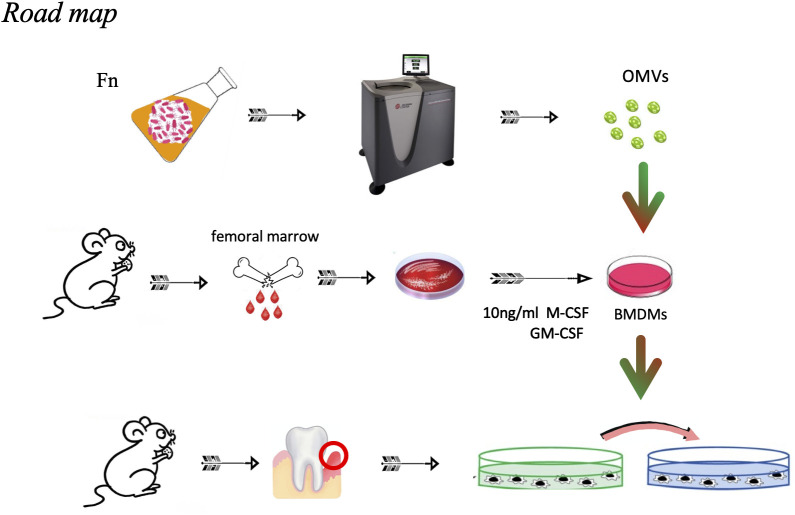

## Introduction

Periodontitis is a periodontal inflammatory disease caused by bacteria, and numerous pathogenic microorganisms destroy the immune defense system in periodontal tissue (Kaan et al., 2021; Szafranski et al., 2021; Wade, 2021). Proinflammatory cytokines produced by immune cells can amplify the inflammatory cascade in surrounding tissues, eventually leading to tissue destruction and tooth loss. At present, intervention measures for periodontitis treatment can only alleviate the intensity and progression of the disease and cannot completely cure it (Myneni et al., 2020; Sczepanik et al., 2020; Feres et al., 2021). Due to the complexity of the disease, the pathophysiological mechanism of periodontitis is not clear, and studying its pathogenesis is therefore necessary to elucidate new economic and effective treatments.

*Fusobacterium nucleatum*, a common opportunistic pathogen in oral microorganisms, is known to play an important role in the occurrence and development of periodontitis (Brennan and Garrett, 2019). This bacterium participates in plaque biofilm formation, bacterial colonization and mixed infections and can activate a variety of signaling pathways and regulate the biological functions of cells by binding to specific adhesins on corresponding cell ligands. *F. nucleatum* can also invade the oral cavity, colon, placental epithelial cells, immune cells, and keratinocytes, among other regions and tissue types. Internalized bacteria affect the synthesis and secretion of some cytokines, regulate biological behaviors such as cell proliferation and apoptosis, and lead to cell dysfunction.

Outer membrane vesicles (OMVs), which are considered to be ubiquitous across all areas of life, are produced by foaming of the bacterial membrane and can include all internal bacterial cell components (nucleic acids, proteins, lipids, and sugars) (Boopathi et al., 2021; Li C. et al., 2021). *F. nucleatum* OMVs are double-layered spherical membrane vesicles that are continuously secreted from the cell surface. Due to the protection provided by the vesicle membrane structure, pathogenic factors at high concentrations can prevent degradation, enter the host cytoplasm through endocytosis, transfer toxic components to host cells, and activate potential proinflammatory or necrotic pathways (Briaud and Carroll, 2020; Engevik, 2020). Therefore, *F. nucleatum* OMVs may be more toxic than the mother bacterium itself.

Macrophages play an important role in the immune response to periodontitis by controlling the pathogenicity of periodontal biofilms and activating the adaptive immune response (Almubarak et al., 2020; Galli and Saleh, 2020; Wang et al., 2021). As we all know, macrophages in a ground state of M0 can be driven to either M1 or M2 states of activation following exposure to external stimulation (Russell et al., 2019). M1 macrophages produce proinflammatory cytokines and osteoclasts, which aggravate the symptoms of periodontitis. M2 macrophages produce anti-inflammatory cytokines to combat periodontitis (Wu et al., 2020; Zhang B. et al., 2021). However, the effects of *F. nucleatum* extracellular vesicles on macrophage polarization in periodontal tissue and their roles in the development of periodontitis have not been reported.

Herein, we aimed to study the interaction between *F. nucleatum* OMVs and macrophages in subjects with periodontitis. In the ligation-induced periodontitis mouse model, the disease status, inflammation and alveolar bone loss caused by *F. nucleatum* OMVs were analyzed. The phenotypic changes and secreted cytokines of macrophages treated with *F. nucleatum* OMVs *in vitro* were analyzed. In the coculture model, the effects of *F. nucleatum* OMVs on the biological function of MGFs were observed.

## Results

### Characteristics of *Fusobacterium nucleatum* Outer Membrane Vesicles

**FIGURE 1:**
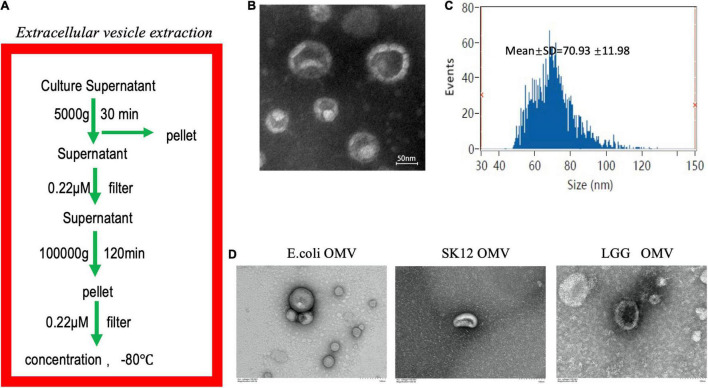
Extraction and identification of *F. nucleatum* OMVs. **(A)** Extraction of *F. nucleatum* OMVs by ultracentrifugation followed these steps. **(B)** *F. nucleatum* OMVs were observed by TEM and exhibited a similar tray structure. **(C)** NTA showed that the OMVs ranged in size from 62–310 nm, with a peak at 131 ± 25 nm. **(D)** The OMVs of *E. coli*, *S. salivarius* K12 and *L. rhamnosus* GG were extracted *via* a similar method and showed different morphologies.

Outer membrane vesicles were first isolated from *F. nucleatum* liquid medium by gradient centrifugation (Figure 1A), and their morphology was observed by transmission electron microscopy (TEM) (Figure 1B). Nanoparticle tracking analysis (NTA) showed that the OMVs ranged in size from 62–310 nm, with a peak at 131 ± 25 nm (Figure 1C). SDS-PAGE revealed that the *F. nucleatum* OMVs had a molecular weight of approximately 40 kD (Supplementary Figure 1A). Mass spectrometry identified the main component as a major outer membrane protein (OMP) that plays roles in (1) immune regulation and (2) adhesion and (3) serves as a channel protein (Supplementary Figure 1B). The OMVs of *Escherichia coli* (*E. coli*), *Streptococcus salivarius* (*S. salivarius*) K12 and *Lactobacillus rhamnosus* (*L. rhamnosus)* GG were also extracted and subsequently used as experimental controls (Figure 1D).

### *Fusobacterium nucleatum* Outer Membrane Vesicles Induce Significant Proinflammatory Profiles and Oxidative Stress in Macrophages

**FIGURE 2:**
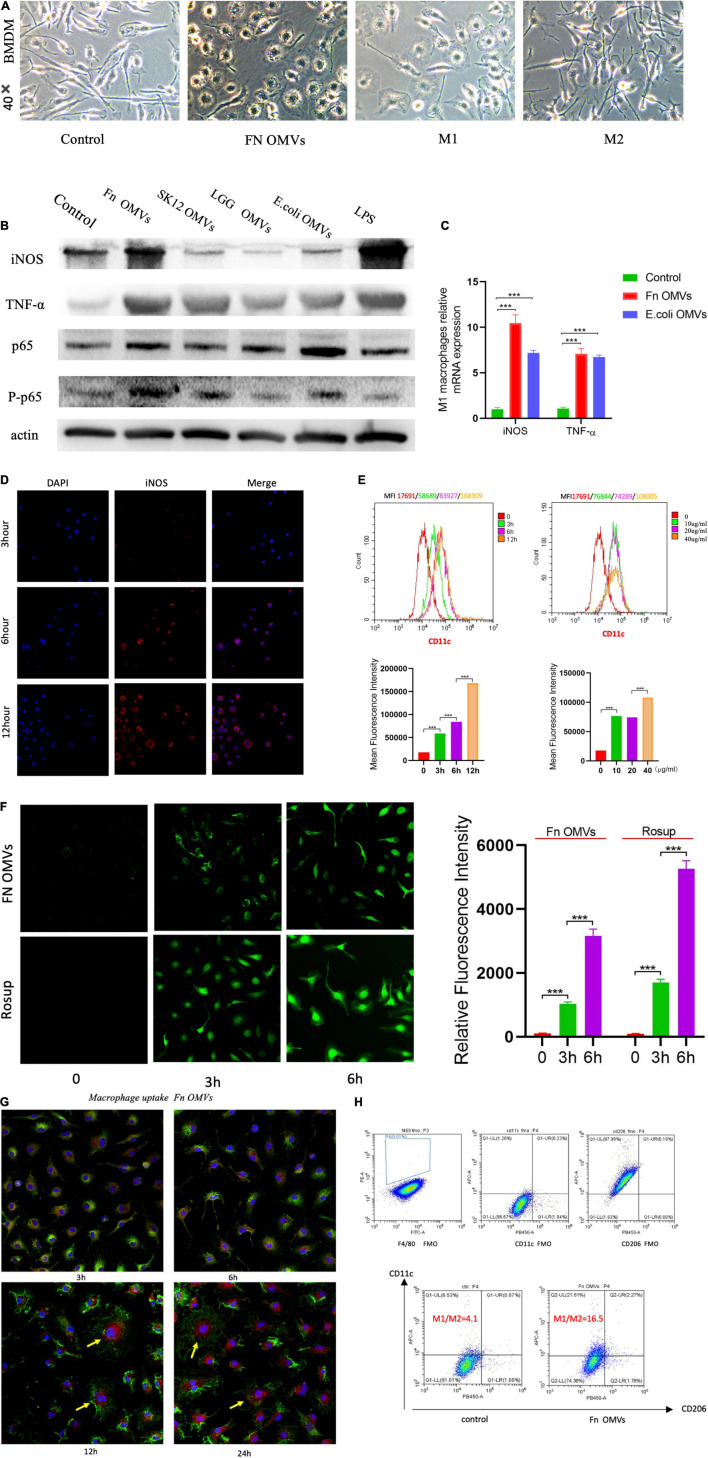
*Fusobacterium nucleatum* OMVs induce significant proinflammatory profiles and oxidative stress in macrophages. **(A)** BMDMs were stimulated separately with *F. nucleatum* OMVs (1.0 μg/ml), M1 macrophages (100 ng/ml) together with IFN-γ (50 ng/ml) or M2 macrophages (10 ng/ml) together with IL-13 (10 ng/ml) for 24 h. BMDMs were stimulated with *F. nucleatum* OMVs for 24 h and were morphologically similar to M1 macrophages. **(B)** *F. nucleatum* OMVs upregulated the expression of the cytokines TNF-α and iNOS, M1 macrophage markers. The expression of P-p65 was increased in macrophages, indicating that the OMVs activated the NF-κB pathway. **(C)** The mRNA level of TNF-α and iNOS increased significantly after OMVs stimulation for 24 h. **(D)** The fluorescence intensity of the M1 biomarkers iNOS were brightest in BMDMs stimulated with *F. nucleatum* OMVs (10 μg/ml) for 12 h. **(E)** Flow cytometry results showed that the expression of CD11c was most expression at *F. nucleatum* OMVs stimulation for 12 h. Furthermore, after more OMVs stimulated BMDM, CD11c expression increased. **(F)** The ROS levels were increased in macrophages stimulated with *F. nucleatum* OMVs for 3 h. ROS staining was brighter at 6 h than at 3 h. **(G)** Macrophages exhibited an morphology after being stimulated for 12 h. *F. nucleatum* OMVs were phagocytosed into cells and appears to surround cell nucleus. (yellow: cell nuclei; red: OMVs; green: membrane). **(H)** After the stimulation of RAW264.7 cells with *F. nucleatum* OMVs for 24 h, flow cytometry analysis revealed that the M1/M2 cell ratio was increased by fourfold compared with that in the control group. *P < 0.05, **P < 0.01, ***P < 0.001. FMO controls are as followed: F4/80 FMO control cells stained with FITC, BV 421, and APC conjugated antibodies. CD206 FMO control cells stained with FITC, PE, and APC conjugated antibodies. CD11c FMO control cells stained with FITC, PE, and BV 421 conjugated antibodies.

Macrophages play an important role in innate immunity. To study the role of *F. nucleatum* OMVs in inducing the production and secretion of cytokines in macrophages, bone marrow cells were isolated from healthy C57BL/6 mice and then induced to differentiate into BMDMs. The BMDMs were stimulated with *F. nucleatum* OMVs (1.0 μg/ml) for 24 h and were morphologically similar to M1 macrophages (Figure 2A). We next detected the cytokines TNF-α and inducible nitric oxide synthase (iNOS) as well as the phosphorylation of p65 in macrophages stimulated with *F. nucleatum* OMVs (Figure 2B). The mRNA level of iNOS and TNF-α was increased significantly after *F. nucleatum* OMVs or *E. coli* OMVs stimulation (Figure 2C). Arg-1 and CD163 are all M2 macrophages biomarkers. The expression of protein and mRNA of Arg-1 and CD163 did not change (Supplementary Figures 2A,B). However, the protein expression of CD86 increased significantly. Enzyme-linked immunosorbent assay (ELISA) analysis of M1 marker (TNF-α) and M2 marker (IL-10) in cultured supernatant of macrophages treated with OMVs for 24 h showed that TNF-α release increased significantly (Supplementary Figure 2C).

Immunofluorescence analysis showed that the expression of iNOS increased in macrophages as the *F. nucleatum* OMVs stimulation time was extended (Figure 2D). The expression of CD11c was time-dependent and dose-dependent (Figure 2E), suggesting that the *F. nucleatum* OMVs induced the polarization of M0-like macrophages toward the proinflammatory M1-like phenotype. Considering the involvement of reactive oxygen species (ROS) in macrophage polarization, we estimated the ROS levels at different time points of macrophage activation, revealing that the level increased as the stimulation period extended (Figure 2F). At 12 h after the phagocytosis of *F. nucleatum* OMVs, the macrophage morphology was altered (Figure 2G), and the cells released more cytokines (Supplementary Figure 2D). Flow cytometry analysis of RAW264.7 cells stimulated with *F. nucleatum* OMVs for 24 h showed that the M1/M2 cell ratio was increased by fourfold (Figure 2H). Overall, these results suggest that *F. nucleatum* OMVs promote macrophage polarization toward a proinflammatory phenotype and increase cytokine release and oxidative damage.

### *Fusobacterium nucleatum* Outer Membrane Vesicles and M1 Macrophages Induce Mouse Gingival Fibroblasts Death in the Proinflammatory Microenvironment

**FIGURE 3:**
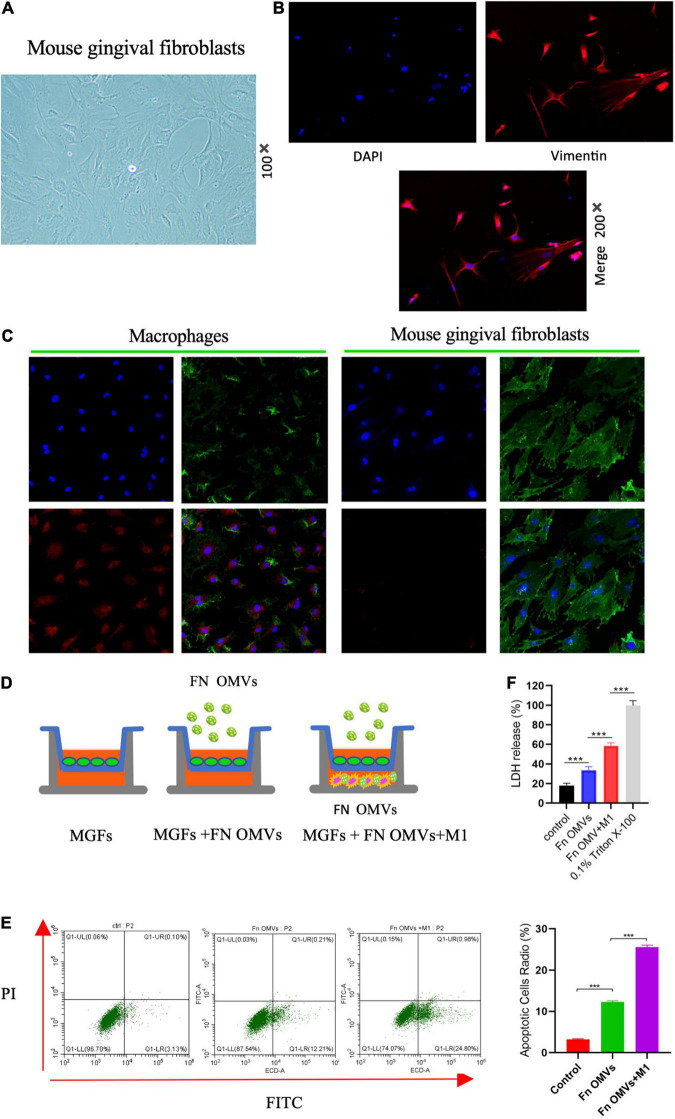
*Fusobacterium nucleatum* OMVs and M1 macrophages induce MGF death in the proinflammatory microenvironment. **(A)** MGFs were cultured, and they exhibited a slender, fibrous morphology (100×). **(B)** Vimentin was expressed at high levels in the cytoplasm. **(C)** Confocal microscopy showed that macrophages engulfed numerous *F. nucleatum* OMVs within 12 h, while almost no *F. nucleatum* OMVs were observed in MGFs within 24 h.(Blue: cell nucleus; Green: cell membrane; Red, Fn OMVs). **(D)** The macrophage/MGF coculture model simulated the interaction between macrophages and *F. nucleatum* OMVs *in vivo*. **(E)** Flow cytometry showed that *F. nucleatum* OMVs alone increased the apoptosis of MGFs after 24 h of treatment. *F. nucleatum* OMVs and M1 macrophage treatment induced more severe apoptosis than that observed in the control group. **(F)** *F. nucleatum* OMVs stimulated the secretion of LDH by MGFs. *F. nucleatum* OMVs and M1 macrophages in combination further exacerbated the release of LDH in MGFs. 0.1% Triton X-100 was used as positive control. *P < 0.05, **P < 0.01, ***P < 0.001.

To simulate the effect of *F. nucleatum* OMVs on macrophages and GFs in periodontal tissue, MGFs were cultured (Figure 3A), and vimentin (Figure 3B) and FSP-1 (Supplementary Figure 3) was shown to be expressed at high levels in the cells. As revealed by confocal microscopy, macrophages engulfed numerous *F. nucleatum* OMVs within 12 h, while almost no *F. nucleatum* OMVs were detected in MGFs within 24 h, and the adhesion between *F. nucleatum* OMVs and the MGF cell membrane was not firm (Figure 3C). Therefore, we established a macrophage/MGF coculture model in a Transwell chamber to simulate the effects of inflammatory factors released by macrophages and *F. nucleatum* OMVs on MGFs (Figure 3D). After 24 h of treatment, flow cytometry showed that *F. nucleatum* OMVs alone were sufficient to affect the apoptosis of MGF cells. *F. nucleatum* OMVs and M1 macrophage treatment induced more severe apoptosis than that observed in the control group (Figure 3E). The level of LDH in culture medium is an indirect indicator of cell damage, and *F. nucleatum* OMVs were shown to stimulate the secretion of LDH from mouse MGFs. The combined use of *F. nucleatum* OMVs and M1 macrophages further exacerbated the release of LDH from MGFs (Figure 3F), indicating that the inflammatory environment further aggravates the toxicity of *F. nucleatum* OMVs on MGFs.

### *Fusobacterium nucleatum* Outer Membrane Vesicles Aggravate Periodontitis in a Mouse Model

**FIGURE 4:**
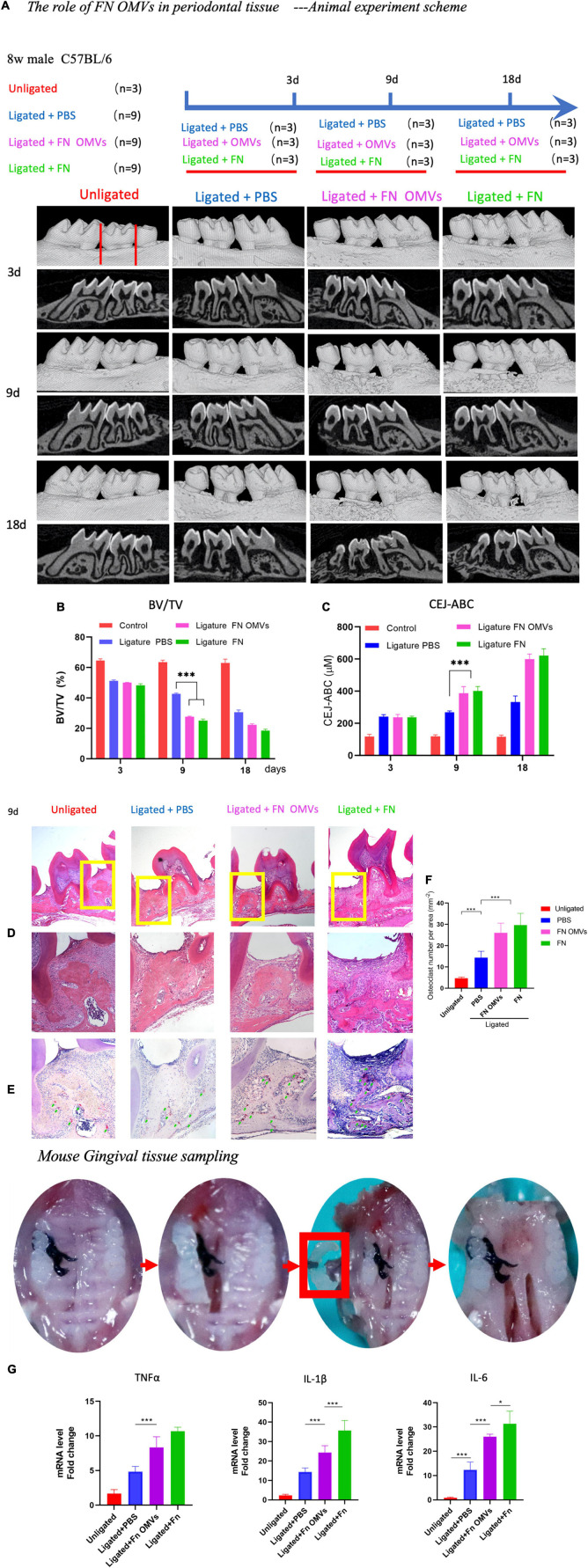
*Fusobacterium nucleatum* OMVs aggravate periodontitis and alveolar bone loss in a mouse model. **(A)** The periodontitis mouse model was constructed, and the mice were divided into four experimental groups: a non-ligation group, ligation + PBS group, ligation + *F. nucleatum* OMV group and ligation + *F. nucleatum* group. The detailed scheme is shown in the section “Materials and Methods”. **(B,C)** Micro-CT images of the mouse maxillas showed more extensive alveolar bone loss and reductions in alveolar bone density. Quantitative analysis of the BV/TV **(B)** and CEJ-ABC distances **(C)** as determined by micro-CT. **(D–F)** Histological images of H&E and TRAP staining. The extent of alveolar bone loss in periodontal tissue **(D)** and quantitative analysis of osteoclasts **(E,F)** are shown. **(G)** Expression of inflammatory factors (IL-1β, IL-6, and TNF-α) in corresponding mouse gingival tissues. The data are presented as the mean ± SD. Significant differences between the groups, *P < 0.05, **P < 0.01, ***P < 0.001. CEJ, cement-enamel junction; ABC, alveolar bone crest; BV/TV, bone volume/total volume.

To observe the effects of *F. nucleatum* OMVs *in vivo*, a mouse model of periodontitis was constructed, and the mice were divided into four experimental groups: a non-ligation group, ligation + PBS group, ligation + *F. nucleatum* OMV group and ligation + *F. nucleatum* group (Figure 4A). Compared with those of mice in the simple ligation group, the maxilla of *F. nucleatum* OMV-treated mice showed more extensive alveolar bone loss and a twofold reduction in alveolar bone density as determined by micro-CT analysis (Figure 4B). At week 9, the cement-enamel junction-alveolar bone crest (CEJ-ABC) distance in the *F. nucleatum* OMV treatment group was greater than that in the simple ligation group. The CEJ-ABC distance in mice treated with *F. nucleatum* OMVs was 1.3-fold higher than that in mice of the simple ligation group (Figure 4C). H&E staining showed that the degree of alveolar bone loss in the periodontal tissues of mice treated with *F. nucleatum* OMVs was more extensive than that in the tissues of mice from the simple ligation group. The periodontal tissue and alveolar bone were intact in the non-ligation group (Figure 4D). The results of the histological analyses of periodontal tissue destruction and alveolar bone loss support the results of the micro-CT analysis. Alveolar bone loss is induced by the osteoclast activity being increased in subjects with periodontitis, and the TRAP staining of tissue sections revealed a significant increase in the number of osteoclasts in the *F. nucleatum* OMV treatment group (Figure 4E). Quantitative analysis of osteoclasts in the tissue sections revealed that the number of osteoclasts in *F. nucleatum* OMV-treated mice was more than twofold higher than that in control mice (Figure 4F). The expression of inflammatory factors (IL-1β, IL-6, and TNF-α) in the corresponding gingival tissues differed between the *F. nucleatum* OMV and PBS treatment groups (Figure 4G). In conclusion, the *in vivo* experiments revealed that the periodontitis in *F. nucleatum* OMVs + ligation group mice was more severe than that of mice in the PBS + ligation group and that *F. nucleatum* OMVs promoted the occurrence and development of periodontitis.

## Discussion

As a periodontal pathogen, *F. nucleatum* plays an indispensable role in the formation and growth of oral biofilms, affects the occurrence and development of periodontitis and is the main microorganism of biofilms in periodontal pockets (Thurnheer et al., 2019; Curtis et al., 2020). Advances in research have revealed an increasing number of virulence factors (Ellis and Kuehn, 2010; Zanzoni et al., 2017), and known virulence factors, new functions and mechanisms are continuously being reported (Li D. H. et al., 2021; Zhang Z. et al., 2021). In addition to its significant “bridging” characteristics, the adhesin of *F. nucleatum* can bind to non-immune and immune cells, leading to gingival inflammation that subsequently develops into an irreversible periodontal disease. FadA adhesin binds to E-cadherin expressed by epithelial and endothelial cells, and β-catenin then enters the nucleus, activates Wnt signaling pathways and promotes cell proliferation (Rubinstein et al., 2013, 2019). The binding of Fap2 to the gal/galNAc polysaccharide on the cell surface is conducive to the bacterial internalization of non-phagocytic cells (Parhi et al., 2020). In addition to Fap2, the non-specific porin FomA is one of the most highly expressed OMPs, accounting for approximately 30% of such proteins (Nakagaki et al., 2010). In addition, metabolites produced by *F. nucleatum*, such as fatty acids, hydrogen sulfide and endotoxins, enhance the immune response of the host (Tateishi et al., 2012; Kezuka et al., 2018; Dahlstrand Rudin et al., 2021). *F. nucleatum* can also invade and reside in epithelial cells of the human gastrointestinal tract and promote intestinal inflammation (Richardson et al., 2020). In general, the molecular mechanisms by which *F. nucleatum* interacts with host cells and regulates innate immunity are not well understood.

During the process of infection, the OMVs released by bacteria can bypass the contact between cells so that key substances, such as active compounds and proteins, can pass through and play an important role in intercellular communication. Recent studies have linked OMVs with immune and disease processes. Studies have shown that almost all gram-negative bacteria can activate target cells by secreting OMVs and transferring toxic components (Deo et al., 2020). Depending on the type of target cells, bacterial species and number of OMVs, the bacterial-host interactions mediated by OMVs can cause non-immunogenic, inflammatory and/or cytotoxic reactions. OMVs can function independently of bacteria, mediate intestinal epithelial reactions and inflammation, and play a role in the pathogenesis of inflammatory bowel disease (IBD) (Durant et al., 2020; Tulkens et al., 2020). *F. nucleatum* secretes OVMs carrying a variety of harmful molecules, which can alter the interactions between microorganisms and the host (Liu J. et al., 2019). *F. nucleatum* OMV stimulation was previously shown to effectively recruit intestinal macrophages, which were differentiated into a proinflammatory phenotype, significantly increase the apoptosis of epithelial cells and cause intestinal barrier dysfunction (Liu et al., 2021). However, whether *F. nucleatum* OMVs also have this function in periodontal tissue has not been reported.

In this study, we first obtained *F. nucleatum* OMVs by ultrafiltration and then determined that they contained pathogenic factors such as *F. nucleatum* autotransporter 2 (Fap2), adhesin a (FadA), and *F. nucleatum* OMP A (FomA) by mass spectrometry analysis (Supplementary Excel file). These pathogenic factors can destroy the biofilm structure and the acid-resistant environment around the teeth, resulting in the occurrence and development of periodontitis (Meng et al., 2021).

Macrophages are key effector cells that protect against heterologous microorganism invasion, mainly killing bacteria through phagocytosis (Locati et al., 2020). Effector cells have phagocytes containing enzymes that can produce ROS and reactive nitrogen species (RNS), and bacteria are phagocytized by macrophages to form phagosomes. They contract in phagosomes and are killed by ROS. Macrophages produce chemokines and inflammatory factors that recruit and activate other immune cells to the site of infection. At the same time, blood monocytes move to the infection site and differentiate into inflammatory macrophages during the anti-infection process (Russell et al., 2019), thus playing a role in defense against bacterial infection.

The effect of *F. nucleatum* OMVs on macrophage polarization is controversial at present. Some researchers believe that they promote macrophage polarization toward the M1 phenotype, while others report that *F. nucleatum* OMVs promote M2 type polarization (Chen et al., 2018; Liu L. et al., 2019; Martin-Gallausiaux et al., 2020; Hu et al., 2021; Liu et al., 2021). We extracted mouse bone marrow mesenchymal cells and induced them to differentiate into macrophages. The cell morphology was altered and the expression of M1 macrophage markers was increased after stimulation by *F. nucleatum* OMVs. Flow cytometry revealed that the expression of CD11c on the macrophage surface was time- and dose-dependent, and ELISA showed that the expression levels of the inflammatory factors IL-1β, IL-6, and TNF-α were increased. Recent studies reported that *F. nucleatum* is a facultative intracellular bacterium that can survive and limit proliferation in macrophages. Live *F. nucleatum* infection can inhibit macrophage apoptosis by activating the PI3K and ERK pathways (Xue et al., 2018; Guo et al., 2020). In conclusion, these data suggest that *F. nucleatum* OMVs promote the transformation of macrophages toward the proinflammatory phenotype and increase the release of corresponding cytokines.

Gingival fibroblasts, the main cell type of periodontal connective tissue, have no tolerance for bacterial stimulation and can continuously respond to exogenous stimulation. The pathogenic effect of *F. nucleatum* on GFs and the possible mechanism have not been fully clarified (Ebersole et al., 2020). *F. nucleatum* can inhibit the proliferation of GFs by regulating the Akt/MAPK pathway. *F. nucleatum* also activates the NF-κB signaling pathway, promotes the production of ROS and the secretion of inflammatory cytokines, and then promotes the apoptosis of GFs (Kang et al., 2019b). Of course, these studies assessed the direct interaction between *F. nucleatum* OMVs and GFs. When *F. nucleatum* OMVs enter periodontal tissue *in vivo*, immune cells first play an immune defense role; for example, macrophages phagocytize OMVs to resist GF damage. After the treatment of MGFs with *F. nucleatum* OMVs for 24 h, no adhesion or invasion was observed between the OMVs and MGFs. After 12 h, numerous OMVs were phagocytosed by the macrophages, which exhibited an altered cellular morphology. Therefore, we established a macrophage/GF coculture model and found that *F. nucleatum* OMVs increased the GF necrosis in the inflammatory microenvironment. *F. nucleatum* OMVs are thought to promote the M1 polarization of macrophages and the release of cytokines to induce the formation of an inflammatory microenvironment in the surrounding tissues, which increases the extent of MGF damage induced by *F. nucleatum* OMVs.

### Limitations of This Study and Follow-Up Research Directions

1. In this study, the effect of *F. nucleatum* OMVs on mouse periodontal tissue was observed and compared with those of PBS and *F. nucleatum*. Previous animal experiments suggested that *F. nucleatum* and *P. gingivalis* in combination promote periodontitis. However, our *in vivo* experimental results show that only *F. nucleatum* OMVs or *F. nucleatum* cause periodontitis, and Meng et al. (2021) reported similar results. They found that *F. nucleatum* produced amyloid-like FadA as a scaffold for biofilm formation, conferred cells with acid tolerance, and mediated its binding to host cells. Amyloid-like FadA was shown to induce periodontal bone loss and promote colorectal cancer (CRC) progression in mice (Meng et al., 2021). Other research teams also found that *F. nucleatum* could inhibit cell proliferation, promote cell apoptosis, and elevate pro-inflammatory cytokine production of osteoblasts or Gingiva-Derived Mesenchymal Stem Cells (Kang et al., 2019a; Gao et al., 2020). More experiments are needed to support this conclusion in the future.

2. Further verification *in vivo* is needed. For example, by knocking out the expression of FadA in *F. nucleatum*, scientists might determine whether *F. nucleatum* or *F. nucleatum* OMVs can still promote the development of periodontitis. The pathogenic factors of *F. nucleatum* must be identified before clinical transformation can occur.

## Materials and Methods

### Bacteria and Cell Culture

*Fusobacterium nucleatum* ATCC 25586 was grown anaerobically (10% CO_2_, 10% H_2_, and 80% N_2_) on brain heart infusion (BHI) agar plates supplemented with 5% defibrinated sheep blood, 5 μg/mL hemin, 1 μg/mL vitamin K1, and 0.5% yeast extract. The bacteria on the agar plate were scraped into BHI liquid medium and then grown to the logarithmic growth phase prior to being used in experiments. *L. rhamnosus* and *S. salivarius* were purchased from BeNa Culture Collection (Beijing, China), and *E. coli* was provided by the Microbiome Medicine Center of Zhejiang Hospital.

Mouse gingival fibroblasts were purchased from Procell Life Science&Technology Co., Ltd. (Wu Han, China) and cultured in α-MEM (Thermo Fisher Scientific, Waltham, MA, United States) supplemented with 10% fetal bovine serum (Thermo Fisher Scientific, Waltham, MA, United States), 100 U/ml penicillin and 100 μg/ml streptomycin in an incubator with 5% CO_2_ at 37^°^C. When the cells reached 90% confluence, they were digested with 0.25% trypsin and subcultured. Early MGFs were used before the fifth generation, and MGFs were extracted as described previously (Mizraji et al., 2013).

The mouse macrophage cell line RAW264.7 (ATCC TIB-71) was cultured in Dulbecco’s modified Eagle’s medium (DMEM, Thermo Fisher Scientific, Waltham, MA, United States) supplemented with 10% fetal bovine serum (Thermo Fisher Scientific, Waltham, MA, United States), 1% penicillin and streptomycin (Life Technologies, Waltham, MA, United States), and 1% L-glutamine.

### Preparation of Outer Membrane Vesicles From *Fusobacterium nucleatum*, *Lactobacillus rhamnosus*, *Streptococcus salivarius*, and *Escherichia coli*

Briefly, after pelleting the bacterial cultures (5,000 g for 30 min), the obtained supernatants were filtered (0.22 μm) to remove parental bacterial debris and other contaminants. The supernatant was ultracentrifuged at 4°C for 120 min at 100,000 g and washed with PBS twice to obtain the crude OMVs. The purified OMVs were refiltered (0.22 μm) and then subjected to sucrose density gradient ultracentrifugation in a 45 Ti rotor at 100,000 g for 2 h at 4°C. The final pellets were resuspended in PBS and stored at -80°C, and the concentration was determined by a BCA protein analysis kit (KeyGEN BioTECH Co., Nanjing, China).

### Identification and Observation of Outer Membrane Vesicles

The extracted OMVs were collected in tubes, and their diameters and particle numbers were measured by NTA (Particle Metrix Co., Germany). In addition, the OMVs were fixed in 2.5% glutaraldehyde, dehydrated in a series of acetone and embedded. The samples were sliced into thin sections and morphological observed on a transmission electron microscope (HT7700, Hitachi, Japan).

### Isolation of Bone Marrow-Derived Macrophages

Bone marrow cells were collected from the femurs and tibias of C57BL/6 wild-type mice (6 weeks old) and cultured as previously described (Ying et al., 2013). Briefly, the cells were differentiated into BMDMs in DMEM (Thermo Fisher Scientific, Waltham, MA, United States) supplemented with 10% fetal bovine serum (Thermo Fisher Scientific, Waltham, MA, United States) and antibiotics in the presence of 25 μg/ml macrophage colony-stimulating factor (M-CSF; R&D Systems, Minneapolis, MN, United States) for 7 days in a 37°C incubator.

### Flow Cytometry

The expression of CD11b, CD206, CD11c, and F4/80 in BMSC-derived macrophages were analyzed using the FACS Calibur (Becton Dickinson, United States) and the CellQuest software (Becton Dickinson). The adherent cells were washed with PBS and collected using Accutase (Nacalai Tesque, Kyoto, Japan) and resuspended in 50 μL staining buffer (BD Pharmingen, Franklin Lakes, NJ, United States). The harvested cells were blocked with 2 μL Human Trustain FcX (Fc receptor Blocking Solution; BioLegend) for 10 min at room temperature, and stained with 2 μL antibodies in the dark for 30 min at 4°C. FITC anti- mouse CD11b, FITC anti-human CD11b, Alexa Fluor^®^488 anti-human CD11b, BV421 anti-mouse CD206, PE anti-mouse F4/80 and APC anti-mouse CD11c (BioLegend).

### Enzyme-Linked Immunosorbent Assay

The ELISA was performed to measure the expression levels of proteins in periodontal tissue extracts and conditioned culture medium of mouse macrophages. The periodontal tissue cell lysate was prepared as described previously (Marchesan et al., 2018). The concentrations of the proinflammatory cytokines TNF-α, IL-6, and IL-1β in periodontal tissue extracts and cell culture supernatants were measured using a Mouse ELISA Standard Kit (Cusabio, Wuhan, China) according to the manufacturer’s protocol.

### Measurement of Reactive Oxygen Species

Reactive oxygen species levels were detected by a DCFH-DA probe (Beyotime, Shanghai, China). After incubation with a ROS staining solution at 37°C, macrophages were washed three times with PBS, and H_2_O_2_ served as the reference. Fluorescence was detected by a fluorescence microplate reader (Olympus, Tokyo, Japan).

### Lactate Dehydrogenase Release Assay

The LDH levels of MGFs were measured following 24 h of co-culture using LDH activity kits (cat. no. A020-2-2; Jiancheng, Nanjing, China) according to the manufacturer’s protocol. The absorbance of each well was measured at 450 nm to determine the LDH activity.

### Development of the Ligation-Induced Periodontitis Mouse Model

A total of 30 wild-type male C57BL/6 mice (6 weeks old, male, 20 g body weight) were used in this study. Male C57BL/6 mice were obtained from Guangdong Medical Laboratory Animal Center and bred at the Experimental Animal Research Center of Southern Medical University. The Experimental Animal Ethics Committee of Southern Medical University approved all of the animal care and study protocols (LAEC-2020-224FS). Studies involving animals were performed in compliance with all relevant ethical regulations. For antibiotic treatment, the mice were given a combination of vancomycin (0.5 mg/ml), gentamicin (1 mg/ml), metronidazole (1 mg/ml), and ampicillin (1 mg/ml) *via* their drinking water for 1 week according to a previous study (Xu et al., 2021).

Ligation-induced periodontitis was induced as described previously (Abe and Hajishengallis, 2013; Pathak et al., 2021). Briefly, the mice were anesthetized by isoflurane and fixed on animal-specific fixed plates in the prone position. The oral cavity was disinfected with 75% ethanol, and a 5-0 silk thread was ligated around the left upper second molar and fixed with a surgical knot. All the knots were placed on the palatal side. After the ligation was completed, the mouse’s tongue was slightly pulled out of the mouth and placed in a warmer place until the mouse woke up. The mice were fed normal chow and had free access to drinking water.

In addition to assuring that the ligation silk line was in place every day, the mice in the PBS, *F. nucleatum* OMV and *F. nucleatum* groups were injected with PBS, *F. nucleatum* OMVs and *F. nucleatum via* the ligation silk line every day. Next, 1 × 10^9^ colony-forming units (CFUs) of *F. nucleatum* or 1 μg/ml *F. nucleatum* OMVs in a 1 ml volume were administered to the maxillary molar teeth *via* the silk threads once every 3 days. If the silk thread was loose or the thread knot was incomplete, the mouse was excluded from the experiment. The day of ligation was considered as day 0. After 3, 9, and 18 days of ligation, the mice were sacrificed by cervical dislocation, and periodontal tissue samples were collected and analyzed to detect pathophysiological changes that were indicative of periodontitis.

### Microcomputed Tomography

Mouse maxillary specimens fixed with paraformaldehyde were scanned by a high-resolution micro-CT (Bruker-Micro-CT, Kontich, Belgium). Data were acquired at 45 keV, with a 10 μm isotropic voxel size. Three-dimensional reconstruction was performed from a set of 400 slices, and the CEJ-ABC was measured from the cementoenamel junction to the ABC. SkyScan Data viewer software was used to measure the buccal and palatal bone resorption heights of the maxillary second molars.

### Histopathological Staining

Paraffin-embedded periodontal tissue sections were sliced at a thickness of 5 μm and then stained with H&E to observe changes in alveolar bone, periodontal ligaments and gingival connective tissue. Images were obtained by microscopy (Olympus, Tokyo, Japan).

### Tartrate Acid Phosphatase Staining

To visualize osteoclasts, tissue sections were stained using a TRAP kit (Servicebio, Wuhan, China). TRAP-stained histological tissue sections were examined under a microscope (Olympus, Tokyo, Japan), and images of predefined areas were captured. The TRAP-positive osteoclasts in the alveolar bone tissue section between the first and second molars were counted.

### RNA Extraction and Quantitative Real-Time PCR Analysis

Total RNA was isolated from cells using TRIzol (Thermo Fisher Scientific, Waltham, MA, United States) according to the manufacturer’s protocol as we previously described (Chen et al., 2020). The total RNA was reverse transcribed into cDNA using Reverse Transcriptase Premix. Quantitative real-time PCR was performed on the ABI vii7 system using primers for the indicated genes and SYBR Green Master Mix according to the manufacturer’s instructions. The relative quantification values for each gene were calculated by the 2^–ΔΔCt^ method, and GAPDH was used as the internal reference. The primers used (Sangon Biotech Co., Ltd., Shanghai, China) are shown in the Supplementary Table.

### Immunoblot Analysis

Cells were lysed in RIPA buffer (150 mM sodium chloride, 1% Triton X-100, 0.1% SDS, 1% sodium deoxycholate, 50 mM Tris–HCl at pH 7.5, and 2 mM EDTA) containing a protease and phosphatase inhibitor cocktail (KeyGEN, Nanjing, China). The cell extracts were mixed with Protein 5× Sample Buffer (Beyotime, Shanghai, China) and boiled for 10 min. The protein extracts were subjected to SDS-PAGE and transferred onto PVDF membranes (Millipore, Burlington, MA, United States). The membranes were blocked with 5% skim milk in Tris-buffered saline supplemented with 0.1% Tween 20 for 1 h at RT and then incubated with the following primary antibodies at 4°C: β-actin (13E5), phospho-NF-κB p65 (Ser536) (93H1), NF-κB p65 (D14E12), and iNOS (D6B6S) (all purchased from Cell Signaling Technology, Danvers, MA, United States). After washing with TBST and incubating for 1 h with anti-mouse IgG (7076) or anti-rabbit IgG (7074) secondary antibodies (Cell Signaling Technologies) at RT, the protein signals were visualized using electrochemiluminescence detection kits (Merck Millipore, Billerica, MA, United States).

### Statistical Analysis

Statistical analysis was performed using Prism (GraphPad Software, v8.4.0.671). The results were appropriately compared by the unpaired *t*-test or ANOVA and are described in each figure legend. All data are presented as the mean ± standard deviation (SD). *p < 0.05 and ^**^p < 0.01 indicated statistical significance, and the statistical significance threshold was set to *P* < 0.05.

## Data Availability Statement

The original contributions presented in the study are included in the article/Supplementary Material, further inquiries can be directed to the corresponding authors.

## Ethics Statement

The animal study was reviewed and approved by the Experimental Animal Ethics Committee of Southern Medical University approved all of the animal care and study protocols (LAEC-2020-224FS).

## Author Contributions

GC completed the purification, analysis, and identification of extracellular vesicles. QS participated in bacterial culture, cell culture, and some testing. QC participated in animal experimental modeling and early coculture of extracellular vesicles and gingival fibroblasts. HZ designed and directed the whole project. All authors reviewed and edited the manuscript.

## Conflict of Interest

The authors declare that the research was conducted in the absence of any commercial or financial relationships that could be construed as a potential conflict of interest.

## Publisher’s Note

All claims expressed in this article are solely those of the authors and do not necessarily represent those of their affiliated organizations, or those of the publisher, the editors and the reviewers. Any product that may be evaluated in this article, or claim that may be made by its manufacturer, is not guaranteed or endorsed by the publisher.

## Supplementary Material

Supplementary Figure 1 **(A)** The molecular weight of *F. nucleatum* OMVs was approximately 40 kD as determined by SDS-PAGE. **(B)** Mass spectrometry revealed the main component to be a major outer membrane protein that plays roles in (1) immune regulation and (2) and adhesion as well as serves as a (3) channel protein.

Supplementary Figure 2 **(A)** M1 macrophages marker (CD86) and M2 macrophages markers (Arg-1 and CD163) were detected by western blot after OMVs or IL-4 + IL-13 for 24 h. IL-4/IL-13 were used for positive control. OMVs could not change the protein expression of M2 macrophages markers. **(B)** The mRNA level of Arg-1 and CD163 was not altered significantly after OMVs or IL-4 + IL-13 stimulation for 24 h. **(C)** ELISA analysis of M1 marker (TNF-α) and M2 marker (IL-10) in cultured supernatant of macrophages treated with OMVs or IL-4 + IL-13 for 24 h. **(D)** Cytokines (IL-1β, TNF-α, and IL-6) secreted by macrophages gradually increased with time. After stimulation with Fn OMVs for 12 h, macrophages secreted the most inflammatory factors.

Supplementary Figure 3 Immunofluorescence analysis showed the expression of fibroblasts markers FSP1 in MGFs.
