# Supplementary figures and images for: Outer Membrane Vesicles From Fusobacterium nucleatum Switch M0-Like Macrophages Toward the M1 Phenotype to Destroy Periodontal Tissues in Mice
Source: Front Microbiol. 2022 Mar 21;13:815638. doi: 10.3389/fmicb.2022.815638 (PMC8981991; doi:10.3389/fmicb.2022.815638)

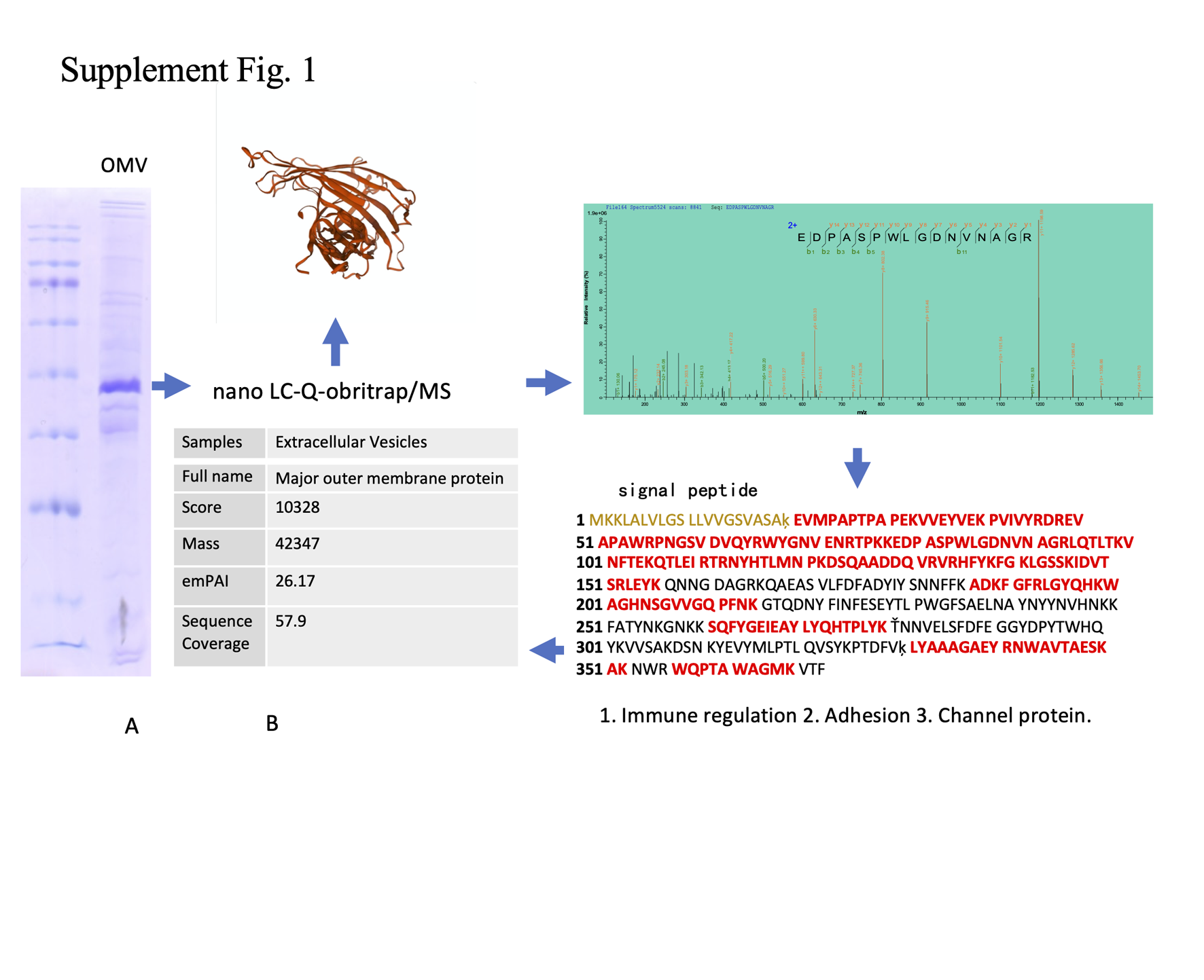

Supplement: Supplementary Figure 1 — (A) The molecular weight of F. nucleatum OMVs was approximately 40 kD as determined by SDS-PAGE. (B) Mass spectrometry revealed the main component to be a major outer membrane protein that plays roles in (1) immune regulation and (2) and adhesion as well as serves as a (3) channel protein. [file Image_1.TIFF]

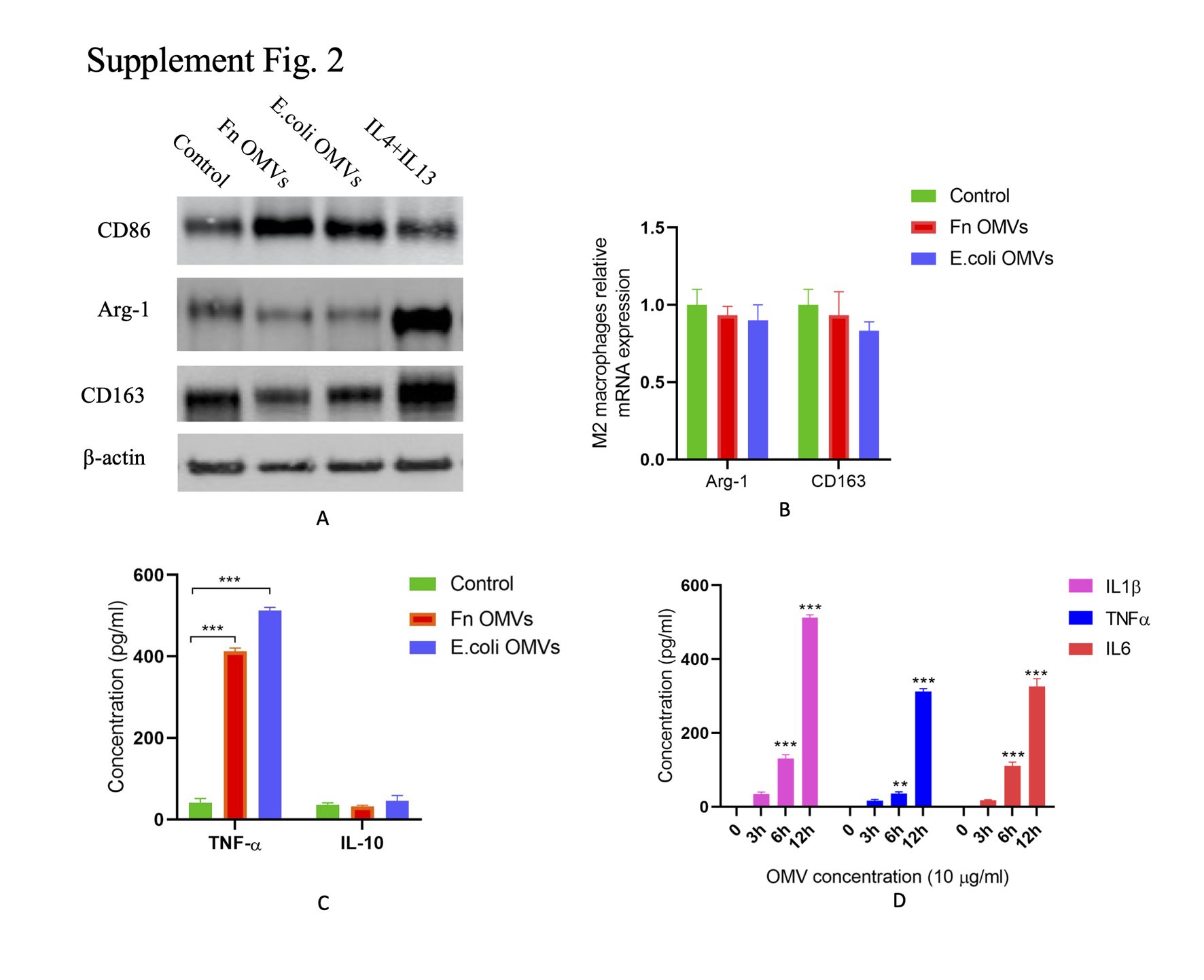

Supplement: Supplementary Figure 2 — (A) M1 macrophages marker (CD86) and M2 macrophages markers (Arg-1 and CD163) were detected by western blot after OMVs or IL-4 + IL-13 for 24 h. IL-4/IL-13 were used for positive control. OMVs could not change the protein expression of M2 macrophages markers. (B) The mRNA level of Arg-1 and CD163 was not altered significantly after OMVs or IL-4 + IL-13 stimulation for 24 h. (C) ELISA analysis of M1 marker (TNF-α) and M2 marker (IL-10) in cultured supernatant of macrophages treated with OMVs or IL-4 + IL-13 for 24 h. (D) Cytokines (IL-1β, TNF-α, and IL-6) secreted by macrophages gradually increased with time. After stimulation with Fn OMVs for 12 h, macrophages secreted the most inflammatory factors. [file Image_2.TIFF]

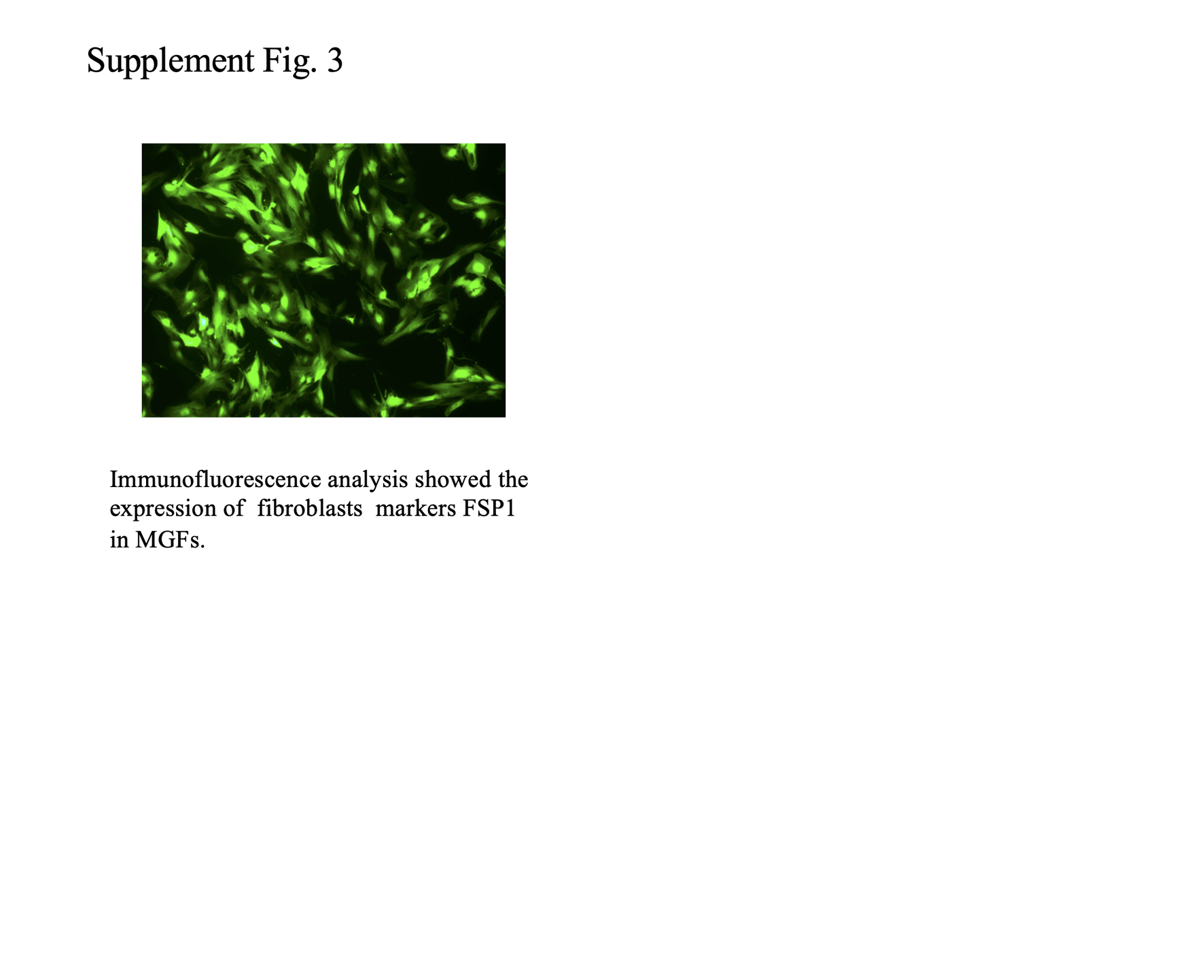

Supplement: Supplementary Figure 3 — Immunofluorescence analysis showed the expression of fibroblasts markers FSP1 in MGFs. [file Image_3.TIFF]
