# Supplementary material for: Outer Membrane Vesicles From Fusobacterium nucleatum Switch M0-Like Macrophages Toward the M1 Phenotype to Destroy Periodontal Tissues in Mice
Source: Front Microbiol. 2022 Mar 21;13:815638. doi: 10.3389/fmicb.2022.815638 (PMC8981991; doi:10.3389/fmicb.2022.815638)
Supplement: Supplementary file 5 [file Table_2.DOCX]

**source data:**

<https://www.jianguoyun.com/p/DQTe3iMQ5fz_CRjHn5oE>
