# Supplementary material for: Outer Membrane Vesicles From Fusobacterium nucleatum Switch M0-Like Macrophages Toward the M1 Phenotype to Destroy Periodontal Tissues in Mice
Source: Front Microbiol. 2022 Mar 21;13:815638. doi: 10.3389/fmicb.2022.815638 (PMC8981991; doi:10.3389/fmicb.2022.815638)
Supplement: Supplementary file 6 [file Table_3.DOCX]

Supplementary Table Primer used for RT-pPCR.

| Gene | Primer sequence (5’–3’ ) |
| --- | --- |
| TNF-α | Forward: CGCTCTTCTGTCTACTGAACTTCGG  Reverse: GTGGTTTGTGAGTGTGAGGGTCTG |
| GAPDH | Forward: GTGAAGGTCGGTGTGAACGG  Reverse: TCCTGGAAGATGGTGATGGG |
| iNOS | Forward: CACCAAGCTGAACTTGAGCG  Reverse: CGTGGCTTTGGGCTCCTC |
| Arg-1 | Forward: AGACAGCAGAGGAGGTGAAGAGTAC  Reverse: AAGGTAGTCAGTCCCTGGCTTATGG |
| CD163 | Forward: GGCTAGACGAAGTCATCTGCAC  Reverse: CTTCGTTGGTCAGCCTCAGAGA |
